# 3D-Printed Devices in Interventional Radiotherapy (Brachytherapy) Applications: A Literature Review

**DOI:** 10.3390/jpm15060262

**Published:** 2025-06-19

**Authors:** Enrico Rosa, Sofia Raponi, Bruno Fionda, Maria Vaccaro, Valentina Lancellotta, Antonio Napolitano, Gabriele Ciasca, Leonardo Bannoni, Patrizia Cornacchione, Luca Tagliaferri, Marco De Spirito, Elisa Placidi

**Affiliations:** 1 UOC Fisica per le Scienze della Vita, Dipartimento di Diagnostica per Immagini e Radioterapia Oncologica, Fondazione Policlinico Universitario A. Gemelli IRCCS, 00168 Rome, Italypatrizia.cornacchione@policlinicogemelli.it (P.C.);; 2 Department of Theoretical and Applied Sciences, eCampus University, 22060 Novedrate, Italy; 3 Dipartimento di Neuroscienze, Sezione di Fisica, Università Cattolica del Sacro Cuore, 00168 Rome, Italy; 4 UOC Degenze di Radioterapia Oncologica, Dipartimento di Diagnostica per Immagini e Radioterapia Oncologica, Fondazione Policlinico Universitario A. Gemelli IRCCS, 00168 Rome, Italy; valentina.lancellotta@policlinicogemelli.it (V.L.); leonardo.bannoni@policlinicogemelli.it (L.B.);; 5 Medical Physics Unit, Bambino Gesù Children’s Hospital, 00165 Rome, Italy; antonio.napolitano@opbg.net; 6 Dipartimento di Scienze Radiologiche ed Ematologiche, Università Cattolica del Sacro Cuore, 00168 Rome, Italy

**Keywords:** interventional radiotherapy, brachytherapy, 3D printing, patient-specific applicator, template

## Abstract

**Introduction**: Interventional radiotherapy (brachytherapy, IRT, BT) has evolved with technological advancements, improving dose precision while minimizing exposure to healthy tissues. The integration of 3D-printing technology in IRT has enabled the development of patient-specific devices, optimizing treatment personalization and dosimetric accuracy. **Methods**: A systematic literature search was conducted in PubMed, Scopus, and Google Scholar to identify studies published between 2020 and 2024 on 3D-printing applications in IRT. The selection process resulted in 74 peer-reviewed articles categorized by radioactive source, brachytherapy technique, endpoint of the 3D-printed product, and study type. **Results**: The analysis highlights the growing implementation of 3D-printed devices in brachytherapy, particularly in gynecological, prostate, and skin cancers. Most studies focus on technique, including intracavitary, interstitial, and contact applications, with custom applicators and templates emerging as predominant endpoints. The majority of studies involved in vivo clinical applications, followed by in silico computational modeling and in vitro experiments. **Conclusions**: The upward trend in scientific publications underscores the growing attention on 3D printing for enhancing personalized brachytherapy. The increasing use of 3D-printed templates and applicators highlights their role in optimizing dose delivery and expanding personalized treatment strategies. The current research trend is shifting toward real-world data and in vivo studies to assess clinical applications, ensuring these innovations translate effectively into routine practice. The integration of 3D printing represents a major advancement in radiation oncology, with the potential to enhance treatment efficacy and patient outcomes. Future research should focus on standardizing manufacturing processes and expanding clinical validation to facilitate broader adoption.

## 1. Introduction

Over time, interventional radiotherapy (brachytherapy, IRT, BT) evolved with the development of safer radioactive sources such as cesium-137, iridium-192, and iodine-125, improving dose control to tumor tissues while minimizing side effects on surrounding healthy tissues. Today, IRT stands as a cornerstone of modern radiation oncology, particularly in the treatment of gynecological, prostate, and skin cancers [1]. A growing body of literature highlights the continuous evolution of IRT, driven by technological advancements and the need for improved treatment personalization. Recent studies demonstrate that the integration of novel materials and advanced imaging techniques has led to superior dosimetric precision and enhanced clinical outcomes in various oncological applications [2].

Today, IRT stands as a cornerstone of modern radiation oncology, particularly using intracavitary, interstitial, and contact techniques, which enable precise and localized dose delivery. Nevertheless, IRT is not yet a universally routine component of clinical practice. While its role is well established—especially in the definitive treatment of gynecological malignancies—it is more selectively applied in specific clinical scenarios, such as irregularly shaped skin tumors or salvage settings, where its dosimetric advantages are particularly valuable. Intracavitary IRT involves placing radioactive sources within natural body cavities, making it a standard approach for treating gynecological malignancies such as cervical and endometrial cancer. Interstitial IRT, on the other hand, involves implanting radioactive sources directly into the tumor or surrounding tissue, providing enhanced dose conformity, and is commonly used for prostate, breast, and soft tissue tumors [3]. Contact IRT delivers radiation through direct contact with the tumor surface, making it particularly effective for treating non-melanoma skin cancers [4].

The adoption of 3D-printing technology in IRT has revolutionized the field by providing unparalleled levels of customization and precision. This approach enables the development of patient-specific devices tailored to individual anatomical and pathological needs, significantly enhancing the efficacy and safety of treatments. This adaptability of 3D-printed devices allows clinicians to address even the most complex anatomical and clinical scenarios, improving therapeutic outcomes and patient comfort. Unlike traditional manufacturing methods, which are often limited in their adaptability to patient-specific anatomical complexities, 3D printing enables the development of personalized devices that optimize dose distribution while reducing radiation exposure to organs at risk (OARs). As highlighted by recent studies, 3D-printed templates and applicators have been shown to improve procedural accuracy, reduce treatment-related complications, and enhance patient comfort compared to conventional approaches [5].

The final product of the 3D-printing process can take various forms, depending on clinical requirements such as applicators, templates, and miscellaneous factors. These applicators can be customized to fit patient-specific anatomical features and are particularly useful in addressing challenges such as unfavorable tumor topographies, narrow anatomical structures, or asymmetrical tumors. By enabling highly conformal dose distributions, 3D-printed solutions enhance tumor control probability and minimize exposure to adjacent OARs.

In gynecological IRT, 3D-printed devices, such as multichannel vaginal cylinders and intrauterine tandems with oblique needle channels, have been instrumental in overcoming the limitations of commercial applicators. These tools facilitate improved dose delivery and ensure better coverage of high-risk clinical target volumes (CTV-HR) [6]. In addition to gynecological applications, 3D printing has been widely adopted in the treatment of superficial malignancies, such as non-melanoma skin cancer (NMSC). Custom contact applicators and molds are fabricated to conform to the patient’s unique skin topography for convex and concave anatomy, reducing air gaps, optimizing dose homogeneity, and ensuring precise positioning. These innovations not only improve treatment accuracy but also enable rapid adjustments to accommodate tumor changes during therapy. Hybrid devices that integrate interstitial and surface techniques further expand the possibilities of personalized IRT.

The use of 3D-printed devices spans a variety of research and clinical contexts. In silico studies leverage computational modeling to evaluate new applicator designs and optimize dosimetry. In vitro studies utilize cell models to test device performance and compatibility. In vivo investigations in animal or human subjects assess the clinical applicability and safety of these innovations. Furthermore, real-life clinical studies have demonstrated the practicality and effectiveness of 3D-printed solutions in routine IRT settings. Whether employed as templates or fully functional applicators, these devices provide the precision and adaptability necessary for modern IRT.

Previous reviews have generally explored the integration of 3D printing into IRT from a broader perspective, focusing on its feasibility and general benefits. In contrast, our review specifically examines the clinical applications of 3D printing in IRT, with a particular emphasis on the final 3D-printed products and the types of studies conducted to validate their use. Over the past decade, numerous investigations have approached this technological advancement using different methodologies, including computational modeling, experimental testing, and clinical validation. These efforts highlight the progressive integration of 3D printing into IRT, demonstrating its ability to address anatomical challenges, improve treatment planning, and enhance the overall quality of care. This review aims to provide a comprehensive overview of these advancements, charting the evolution of 3D printing applications and underscoring its potential to further personalize and optimize treatment strategies in IRT.

This review is structured as follows: Section 2 describes the methodology used to select and classify the studies. Section 3 presents the results, including a quantitative and qualitative analysis of the selected papers. Section 4 discusses the key trends and implications, and Section 5 outlines the conclusions and directions for future research.

## 2. Methods

**Figure 1:**
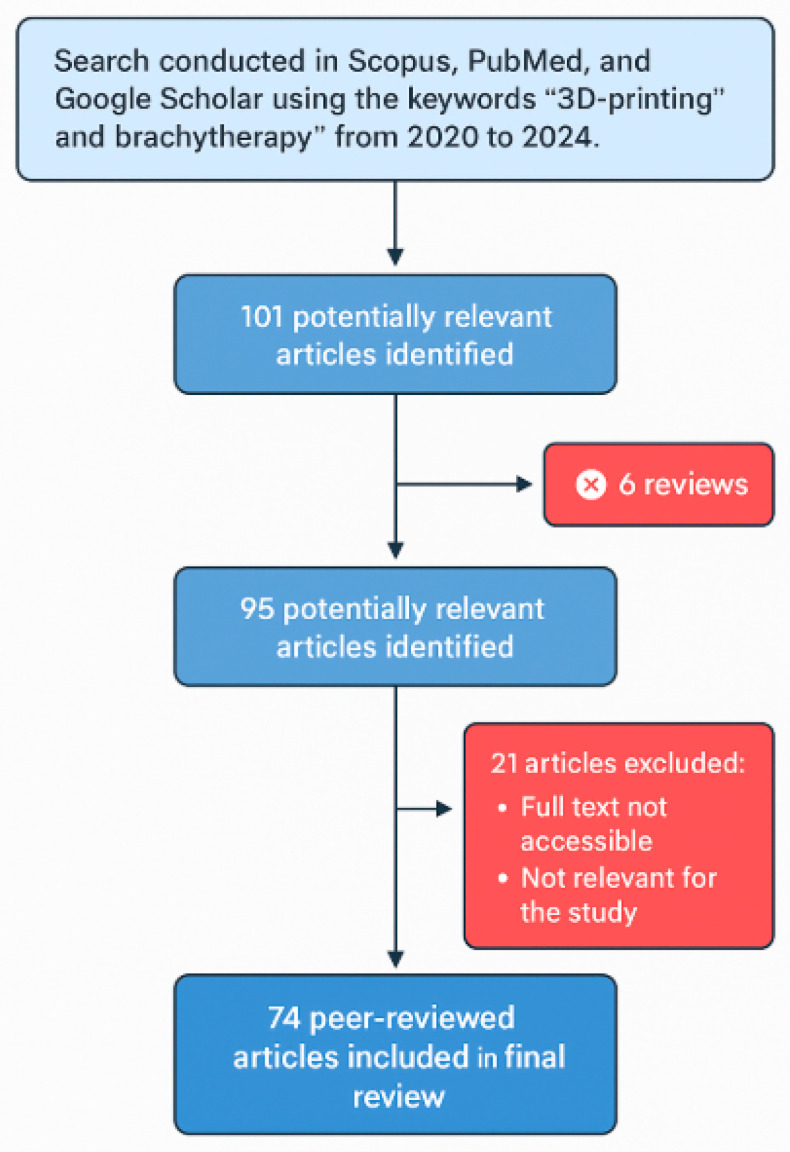
Flowchart of the article selection process for the review on 3D printing in IRT (2020–2024).

This review was conducted by searching the scientific databases PubMed, Scopus, and Google Scholar for studies related to the application of 3D printing in IRT. The keywords “brachytherapy” and “3D-printing” were used to identify relevant publications published between 2020 and 2024. Figure 1 provides a detailed overview of the article selection process.

The initial search yielded 101 potentially relevant articles. Exclusion criteria were then applied to ensure the quality and focus of the review. Specifically, six scientific articles were not considered for the analysis because they were reviews themselves, leaving 95 articles for further evaluation. Subsequently, 21 additional articles were excluded because their full text was inaccessible or deemed irrelevant to the study. This process resulted in 74 peer-reviewed articles that were included in the final analysis.

For each study, information was extracted and categorized based on specific assumptions. Publications where multiple techniques, radioactive sources, or endpoints were reported were considered as distinct entries for each category to allow a detailed evaluation of the various implementations. Endpoints were classified as applicators if the 3D-printed device remains in place during irradiation, containing the catheters or sources within its structure. Templates were defined as devices used solely to geometrically configure the placement of catheters or sources, which are removed before irradiation. Miscellaneous endpoints included other uses, such as devices for quality assurance, immobilization systems, and spacers.

The type of study was categorized based on the methodology used. In vitro studies involved cell cultures, while in vivo studies involved animals or patients. In silico studies included simulations or theoretical investigations. The IRT technique was identified as interstitial, intracavitary, or contact, depending on the specific application.

This structured approach ensured a comprehensive evaluation of advancements in the use of 3D printing within IRT.

## 3. Results

The analysis presented in Table 1 summarizes the characteristics and trends in the application of 3D printing technology in IRT across the 74 included studies. These studies were categorized based on publication year, radioactive source, IRT technique, endpoint of the 3D-printed product, diseases, and type of study. A variety of radioactive sources were identified, including 192Ir, 125I, 60Co, 90Y, and 103Pd, reflecting the range of isotopes commonly used in clinical and experimental settings. The IRT techniques analyzed included interstitial, intracavitary, and contact applications, highlighting the different approaches where 3D printing has been integrated. Endpoints were classified into three main categories: applicators, which remain in place during irradiation; templates, which are used to guide catheter or source positioning and then removed; and miscellaneous applications, encompassing devices for quality assurance, immobilization, or dose modulation. The studies were also categorized by study type, including in vitro experiments on cell cultures, in silico computational simulations, and in vivo investigations involving animal models or patients.

**Figure 2:**
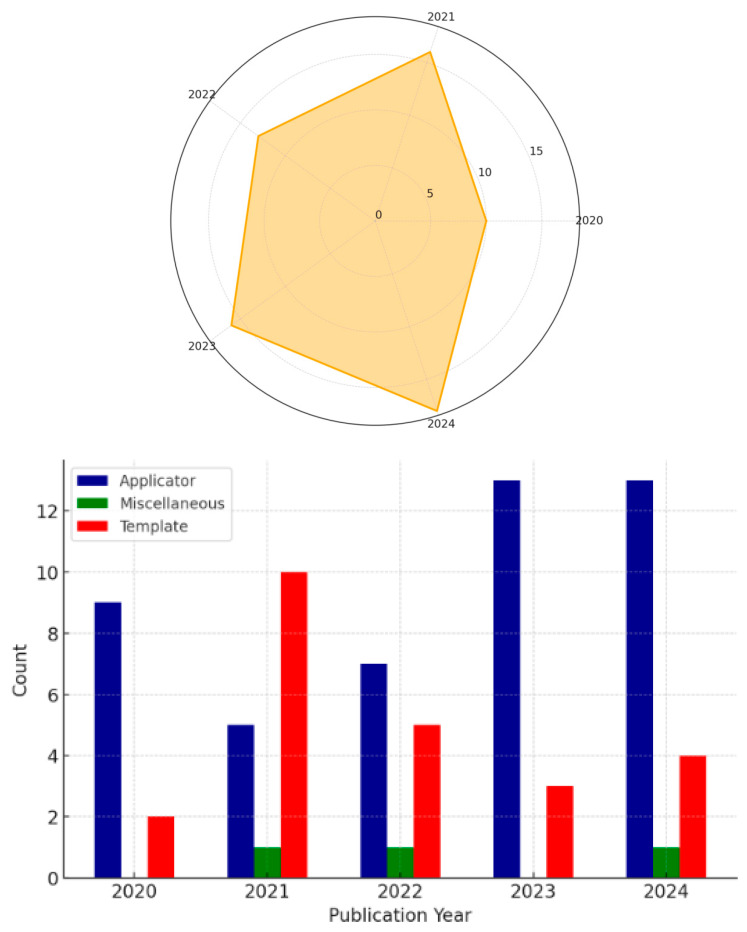
The upper panel presents a radar chart illustrating the distribution of studies across the years from 2020 to 2024, categorized by the total number of studies analyzed. The lower panel displays a bar chart showing the distribution of endpoints (applicator, template, and miscellaneous) per year.

The radar chart (upper panel, Figure 2) shows the yearly distribution of studies, with the highest number of studies observed in 2024 (24.3%), followed by 2021 (21.6%) and 2023 (21.6%), and 2022 (17.6%). The lowest proportion was observed in 2020 (13.5%).

The bar chart (lower panel, Figure 2) illustrates the frequency of endpoints per year. Applicators were the most frequently reported endpoint across all years, with percentages of 81.8% in 2020, 31.3% in 2021, 53.8% in 2022, 81.3% in 2023, and 72.2% in 2024. Templates were the second most reported endpoint, with proportions of 18.2% in 2020, 62.5% in 2021, 38.5% in 2022, 18.8% in 2023, and 22.2% in 2024. Miscellaneous endpoints were rarely reported, with percentages of 0.0% in 2020, 6.3% in 2021, 7.7% in 2022, and 0.0% in 2023 and 5.6% 2024.

**Figure 3:**
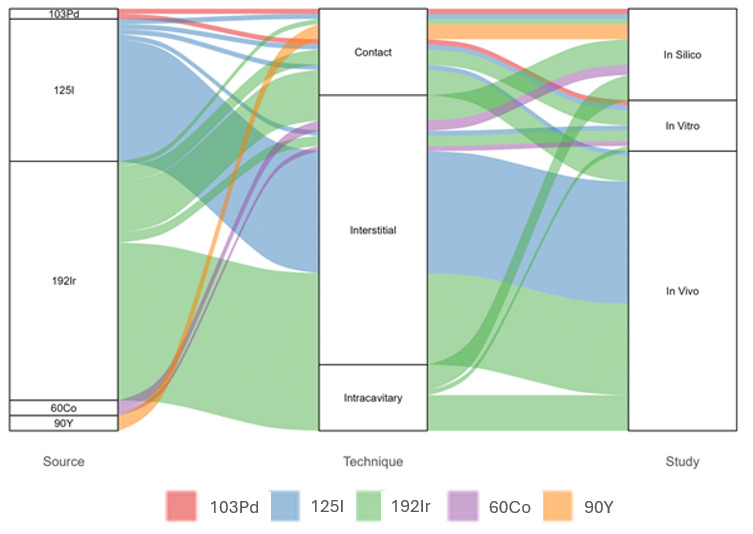
Alluvial plot illustrates the relationships between the radioactive source, technique, and study type in the analyzed articles. The plot highlights the flow of studies from radioactive sources to techniques including contact, interstitial, and intracavitary and subsequently to study types such as in silico, in vitro, and in vivo. The thickness of the flows represents the frequency of occurrences within the dataset.

The alluvial plot (Figure 3) shows that the most frequently used radioactive source is 192Ir, followed by 125I. Interstitial techniques are the most commonly employed, with a significant proportion of studies also using intracavitary and contact techniques. The majority of studies are categorized as in vivo, followed by in silico and in vitro. The plot highlights the connections between sources, techniques, and study types, with 192Ir predominantly associated with interstitial and intracavitary techniques, while 125I is mainly linked to interstitial applications.

**Figure 4:**
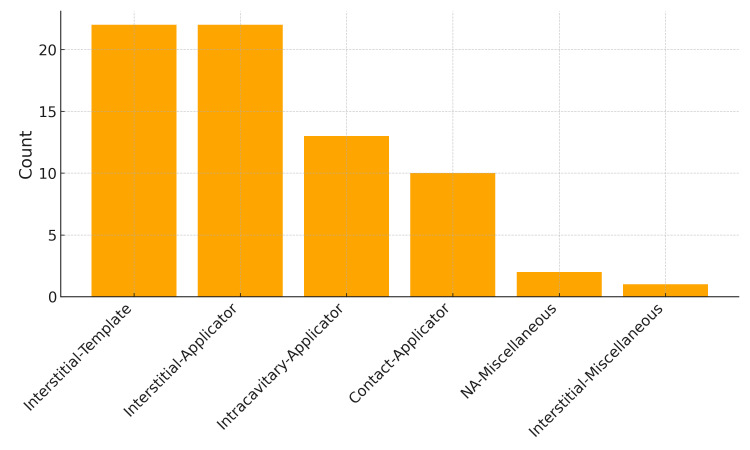
Bar chart presents the distribution of studies categorized by technique and endpoint.

The bar chart (Figure 4) shows that interstitial templates and interstitial applicators are the most frequently reported, each exceeding 20 studies. Intracavitary applicators follow with 13 studies, while contact applicators have 10 studies. Miscellaneous is reported with a lower frequency, at 2 studies, while interstitial miscellaneous has the lowest representation, with only 1 study.

## 4. Discussion

The results highlight the significant role of 3D printing in IRT, with key trends emerging across sources, techniques, endpoints, and study types.

The yearly distribution of articles shows a positive trend overall, with an increase in the number of publications over time. However, there is a noticeable decline in articles published between 2021 and 2022. This deflection might be attributed to challenges in conducting research during the COVID-19 pandemic, which likely impacted the availability of resources and the pace of experimental and clinical studies.

The analysis revealed that 192Ir and 125I are the most frequently used radioactive sources, reflecting their established roles in clinical and experimental settings. 192Ir is the most used source in after-loaders, which further emphasizes its prevalence and versatility in IRT applications. The predominance of 192Ir, particularly in interstitial and intracavitary techniques, underscores its effectiveness for precise dose delivery. In contrast, 125I is primarily linked to interstitial applications, likely due to its low-energy emission characteristics suitable for localized treatments.

Among the techniques, interstitial approaches dominated the dataset, followed by intracavitary and contact techniques. The dominance of interstitial techniques can be attributed to the ability of 3D printing to conform implants more effectively in complex anatomical scenarios where interstitial approaches are preferred. This capability makes interstitial techniques particularly valuable in cases involving irregularly shaped tumors or challenging anatomical regions. Intracavitary techniques, frequently associated with gynecological cancers, also showed strong representation, while contact techniques were more focused on superficial tumors, such as non-melanoma skin cancers.

In terms of endpoints, applicators emerged as the most reported category across all years, highlighting their role as fully functional devices that are ready for use directly in clinical settings. Templates, on the other hand, are closely tied to interstitial techniques in most publications, as they are primarily used to guide catheter or source positioning. This distinction between endpoints reflects the versatility of 3D printing in addressing different clinical needs, with applicators offering a complete solution and templates providing precise guidance for interstitial treatments.

The bar chart further illustrates this distribution, with interstitial applicators and templates being the most frequently reported combinations, exceeding 20 occurrences each. The high frequency of interstitial templates highlights their utility in procedures requiring precise catheter placement in anatomically complex regions. Intracavitary and contact applicators follow, with counts between 10 and 15, reflecting their relevance in specific clinical scenarios, such as gynecological and superficial tumors. Less frequent categories, such as interstitial miscellaneous and intracavitary templates, were reported in fewer than five studies, likely representing specialized or emerging applications of 3D printing in IRT.

Finally, the analysis of study types showed a majority of in vivo investigations. This trend reflects the focus on obtaining results in real-world clinical settings, which often rely on feedback and insights from prior in silico simulations and in vitro experiments using cell cultures. The progression from computational and laboratory-based studies to in vivo applications underscores the strong translational aspect of 3D-printed IRT devices, highlighting their ability to move from experimental development to practical implementation. This translational approach ensures that these technologies are not only innovative but also relevant and impactful in clinical practice.

A major limitation in the current body of evidence is the lack of randomized controlled trials directly comparing 3D-printed moulds with standard devices. As a result, most claims regarding the superiority of 3D-printed solutions are based on theoretical dose distributions or non-randomized feasibility studies. While the preliminary data are promising, these potential advantages require rigorous clinical validation before they can be translated into standardized patient care protocols.

## 5. Conclusions

The integration of advanced technologies, such as 3D printing, is increasingly important in IRT, enabling greater treatment precision, customization, and efficiency. The growing trend in research reflects its expanding role in clinical practice. 3D printing has demonstrated versatility across various radioactive sources, techniques, and endpoints, with interstitial techniques particularly benefiting from its ability to adapt implants to complex anatomical structures. The focus on in vivo studies highlights the translational impact of these technologies, bridging the gap between research and real-world clinical applications. This progression underscores the value of innovative approaches in optimizing and personalizing IRT, paving the way for improved standards of care.

Future research should aim to standardize the design and manufacturing workflows of 3D-printed devices for brachytherapy to ensure reproducibility and quality assurance across institutions. In addition, large-scale prospective clinical trials are needed to validate the clinical efficacy and safety of these technologies. Cost-effectiveness analyses will be critical to assess their sustainability in routine practice. Finally, integrating 3D printing with artificial intelligence-driven planning tools may offer new frontiers in precision oncology and personalized radiotherapy.

## Figures and Tables

**Table 1:** Publication year, radioactive source, IRT technique, endpoint of 3D-printing application, study type, and disease for each analyzed publication.

Publication Year	Source	Technique	Endpoint	Study Type	Disease	Ref
2020	192Ir	Intracavitary	Applicator	In Vitro	Gyn	[7]
2020	192Ir	Interstitial	Applicator	In Vivo	Ocular	[8]
2020	90Y	Contact	Applicator	In Silico	Skin	[9]
2020	125I	Interstitial	Template	In Vivo	Brain	[10]
2020	192Ir	Interstitial	Applicator	In Vivo	Gyn	[11]
2020	192Ir	Intracavitary/Interstitial	Applicator	In Silico	Gyn	[12]
2020	192Ir	Intracavitary	Applicator	In Vivo	Gyn	[13]
2020	125I	Interstitial	Template	In Vivo	Perineal	[14]
2020	125I	Interstitial	Template	In Vivo	Perineal	[15]
2020	192Ir	Interstitial	Applicator	In Vivo	Gyn	[16]
2020	90Y	Contact	Applicator	In Silico	Skin	[17]
2021	192Ir	NA	Miscellaneous	In Vitro	NA	[18]
2021	192Ir	Contact	Applicator	In Vitro	NA	[19]
2021	125I	Interstitial	Template	In Vivo	Ocular	[20]
2021	125I	Interstitial	Template	In Vivo	Gyn	[21]
2021	125I	Interstitial	Template	In Vivo	Thoracic	[22]
2021	192Ir	Intracavitary	Applicator	In Vivo	Gyn	[23]
2021	125I	Interstitial	Template	In Vivo	Gyn	[24]
2021	192Ir	Intracavitary	Applicator	In Silico	Gyn	[25]
2021	125I	Interstitial	Template	In Vivo	Rectal	[26]
2021	125I	Interstitial	Template	In Vivo	Thoracic	[27]
2021	125I	Contact	Applicator	In Silico/In Vitro	Ocular	[28]
2021	60Co	Interstitial	Applicator	In Silico/In Vitro	Gyn	[29]
2021	125I	Interstitial	Template	In Vivo	Gyn	[30]
2021	125I	Interstitial	Template	In Vivo	Thoracic	[31]
2021	125I	Interstitial	Template	In Vivo	Head and Neck	[32]
2021	125I	Interstitial	Template	In Vivo	Rectal	[33]
2022	192Ir	Contact	Applicator	In Vivo	Skin	[34]
2022	125I	Interstitial	Template	In Vivo	Pelvic	[35]
2022	192Ir	Interstitial	Applicator	In Silico	Gyn	[36]
2022	125I	Interstitial	Template	In Vivo	Prostate	[37]
2022	192Ir	Interstitial	Applicator	In Vivo	Gyn	[38]
2022	125I	Interstitial	Miscellaneous	In Vitro/In Vivo	Prostate	[39]
2022	192Ir	Contact	Applicator	In Vivo	Skin	[40]
2022	125I	Interstitial	Template	In Vivo	Pancreatic	[41]
2022	192Ir	Intracavitary/Interstitial	Applicator	In Vivo	Gyn	[42]
2022	192Ir	Intracavitary	Applicator	In Silico	NA	[43]
2022	125I	Interstitial	Template	In Vivo	Esophageal	[44]
2022	192Ir	Contact	Applicator	In Vitro	Head and Neck	[45]
2022	192Ir	Intracavitary	Applicator	In Vivo	Gyn	[46]
2023	192Ir	Interstitial	Applicator	In Vivo	Gyn	[47]
2023	192Ir	Intracavitary	Applicator	In Silico	Gyn	[48]
2023	192Ir	Interstitial	Applicator	In Vitro	Gyn	[49]
2023	192Ir	Contact	Applicator	In Vivo	Skin	[50]
2023	192Ir	Interstitial	Applicator	In Vivo	Gyn	[51]
2023	192Ir	Interstitial	Applicator	In Vitro/In Vivo	Gyn	[52]
2023	192Ir	Interstitial	Template	In Vivo	Gyn	[53]
2023	192Ir	Intracavitary/Interstitial	Applicator	In Vivo	Gyn	[54]
2023	192Ir	Interstitial	Applicator	In Vivo	Gyn	[55]
2023	125I	Interstitial	Applicator	In Vivo	Skin	[56]
2023	192Ir	Intracavitary	Applicator	In Vivo	Gyn	[57]
2023	125I	Interstitial	Template	In Vivo	Sarcoma	[58]
2023	192Ir	Contact	Applicator	In Vitro	Skin	[59]
2023	192Ir/60Co	Interstitial	Applicator	In Silico	Gyn	[60]
2023	125I	Interstitial	Template	In Vivo	Brain	[61]
2023	103Pd	Contact	Applicator	In Silico/In Vitro	Ocular	[62]
2024	192Ir	Interstitial	Applicator	In Silico	Gyn	[63]
2024	192Ir	Interstitial	Template	In Vivo	Head and Neck	[64]
2024	125I	Interstitial	Template	In Vivo	Gyn	[65]
2024	192Ir	Interstitial	Applicator	In Vivo	Gyn	[66]
2024	192Ir	Interstitial	Applicator	In Vivo	Gyn	[67]
2024	192Ir	Intracavitary/Contact	Applicator	In Silico	Gyn	[68]
2024	192Ir	Interstitial	Applicator	In Vivo	Gyn	[69]
2024	192Ir	Interstitial	Applicator	In Vivo	Gyn	[70]
2024	192Ir	Intracavitary/Interstitial	Applicator	In Vivo	Gyn	[71]
2024	125I	Contact	Applicator	In Vivo	Skin	[72]
2024	192Ir	Contact	Applicator	In Vivo	Skin	[73]
2024	192Ir	Interstitial	Applicator	In Vivo	Head and Neck	[74]
2024	125I	Interstitial	Template	In Vivo	Head and Neck	[75]
2024	NA	NA	Miscellaneous	NA	Ocular	[76]
2024	90Y	Contact	Applicator	In Silico	Skin	[77]
2024	192Ir	Contact	Applicator	In Vivo	Skin	[78]
2024	192Ir	Interstitial	Applicator	In Silico	Gyn	[79]
2024	125I	Interstitial	Template	In Vivo	Head and Neck	[80]

## Abbreviations

3D	Three-dimensional
BT	Brachytherapy
CT	Computed Tomography
CTV-HR	High-risk Clinical Target Volume
Gyn	Gynecology
HDR	High Dose Rate
IRT	Interventional Radiotherapy
MRI	Magnetic Resonance Imaging
NA	Not Available
OAR	Organ at Risk
QA	Quality Assurance
